# A novel *ex vivo* Huntington’s disease model for studying GABAergic neurons and cell grafts by laser microdissection

**DOI:** 10.1371/journal.pone.0193409

**Published:** 2018-03-05

**Authors:** E. M. André, N. Daviaud, L. Sindji, J. Cayon, R. Perrot, C. N. Montero-Menei

**Affiliations:** 1 CRCINA, INSERM, Université de Nantes, Université d’Angers, Angers, France; 2 Fishberg Department of Neuroscience and Friedman Brain Institute, Icahn School of Medicine at Mount Sinai, New York, NY, United States of America; 3 PACEM, Angers University, Angers, France; 4 SCIAM, Angers University, Angers, France; University of Florida, UNITED STATES

## Abstract

Organotypic brain slice cultures have been recently used to study neurodegenerative disorders such as Parkinson’s disease and Huntington’s disease (HD). They preserve brain three-dimensional architecture, synaptic connectivity and brain cells microenvironment. Here, we developed an innovative model of Huntington’s disease from coronal rat brain slices, that include all the areas involved in the pathology. HD-like neurodegeneration was obtained in only one week, in a single step, during organotypic slice preparation, without the use of neurotoxins. HD-like histopathology was analysed and after one week, a reduction of 40% of medium spiny neurons was observed. To analyse new therapeutic approaches in this innovative HD model, we developed a novel protocol of laser microdissection to isolate and analyse by RT-qPCR, grafted cells as well as surrounding tissue of fresh organotypic slices. We determined that laser microdissection could be performed on a 400μm organotypic slice after alcohol dehydration protocol, allowing the analysis of mRNA expression in the rat tissue as well as in grafted cells. In conclusion, we developed a new approach for modeling Huntington's disease *ex vivo*, and provided a useful innovative method for screening new potential therapies for neurodegenerative diseases especially when associated with laser microdissection.

## Introduction

Huntington’s disease (HD) is an inherited neurodegenerative disorder due to an increased number of CAG repeats of the huntingtin (*HTT*) gene, leading to a polyglutamine repetition at the NH_2_-terminal of the huntingtin protein (HTT) [1]. This mutation induces a progressive neurodegeneration leading to motor, cognitive and emotional disturbances in patients [2]. Early damages appear in the GABAergic medium spiny neurons (MSNs), which constitute 95% of cells in the striatum [3], disrupting GABAergic projections to the external segment of the globus pallidus (GP) and the substantia nigra *pars reticulata (SNpr)*. Dysfunction of the corticostriatal pathway is also observed in HD and pyramidal cell loss is extensive [4]. Currently, no cure exists to stop or reverse the disease thus; new experimental treatments need to be evaluated [5]. Different models of HD exist. *In vitro* models are developed from primary neuronal cultures or induced-pluripotent stem cells derived from patients [6]. Genetically modified rodents (such as R6/2 mouse) or wild-type rodents treated with specific neurotoxins [7] represent the most common *in vivo* models. Those models were designed to elucidate the pathogenesis, cell death mechanisms and to evaluate therapeutic potential of innovative strategies [8]. However, they require high technical and financial resources, are very time consuming and may raise ethical concerns [9–11].

Organotypic brain slices can be maintained in culture for weeks and provide unique advantages over *in vivo* and *in vitro* platforms [12,13]. They preserve tissue structures, maintain neuronal activities and synapse circuitry, and replicate many aspects of the *in vivo* context [14]. Further advantages of these brain slice cultures may reside in their low-cost, as well as their rapidity and simplicity of use and analysis. Recently, different HD organotypic models have been developed. First, organotypic slices were made directly from transgenic mice expressing HD patterns, such as R6/2 transgenic mice [15,16]. Organotypic slices can also be prepared from wild-type rodents, and GABAergic neuron loss is then obtained by neurotoxin injection, such as kainic acid, quinolinic acid or 3-nitropropionic acid. More recently, a model involving normal slices transfected with HD-polyQ plasmids or with DNA constructs derived from the human pathological *HTT* gene was developed [17–20]. Efficiency of transfection using non-viral vectors remains low, even though biolistics appeared to provide the highest number of positive cells (+/- 34) per slice compared to lipotransfection or electroporation [18] or more recently 35% of the cells in the slice [20]. Viral vectors with pro-aggregant genes of relevance could be also used to create a very nice model of HD, but transduction efficiency is difficult to determine as the transgene is heterogeneously distributed across the slice area. Moreover, 30 days after transduction no cellular apoptosis was yet detected so with that method only the early phase of the disease is modeled [21]

A simple, reproducible model of HD conveniently allowing screening of different therapeutic approaches before moving forward to an *in vivo* model is necessary.

HD is an interesting candidate for stem cell transplantation therapy as it is due to a relatively focal loss of striatal MSNs. It has been shown that transplantation of fetal developing MSNs into the striatum ameliorates motor and cognitive deficits in animal models [22,23]. However, new sources of cells must be found as fetal tissue presents different caveats: scarce tissue, storage and ethical concerns. Thus, grafting of different type of stem cells has been tested as mesenchymal stromal cells or neural stem cells (NSCs) among others [24,25], showing interesting potential for HD cell therapy. To comprehend the mechanisms involved in the therapeutic effect observed with these grafted stem cells, it is essential to analyse their gene expression pattern. However, the reliability of tests based on tissue or cell extracts often depends on the relative abundance of the cell population. In this case, sampling errors or many “contaminating” cells can lead to false negative results. Laser microdissection (LMD) to obtain purified cell populations can overcome this limitation [26,27]. Thus, LMD associated with real-time quantitative PCR (RT-qPCR) has been developed for the analysis of cell-specific gene expression patterns [28]. For instance, LMD has been used to investigate the transcriptional activity of engrafted NSCs and progenitors in mice models of spinal cord injury [29,30].

In this study, we established a novel approach to model HD. We developed a coronal organotypic culture model that includes the principal areas involved in HD in a unique slice. Furthermore, this innovative HD model was obtained without neurotoxins, but due to a mechanical transection of the GABAergic MSN pathway. The viability of this model was assessed and the depopulation of striatal GABAergic MSNs was characterised and quantified over time. We also described here a protocol, which allows studying the messenger ribonucleic acid (mRNA) expression of grafted cells (NSCs here) in a fresh 400μm organotypic slice after isolation by LMD.

## Material and methods

### Ethics

All animal procedures were approved by animal experimentation ethic committee of Pays de la Loire (N°A49-2012- 16).

### Preparation of organotypic slices

**Fig 1:**
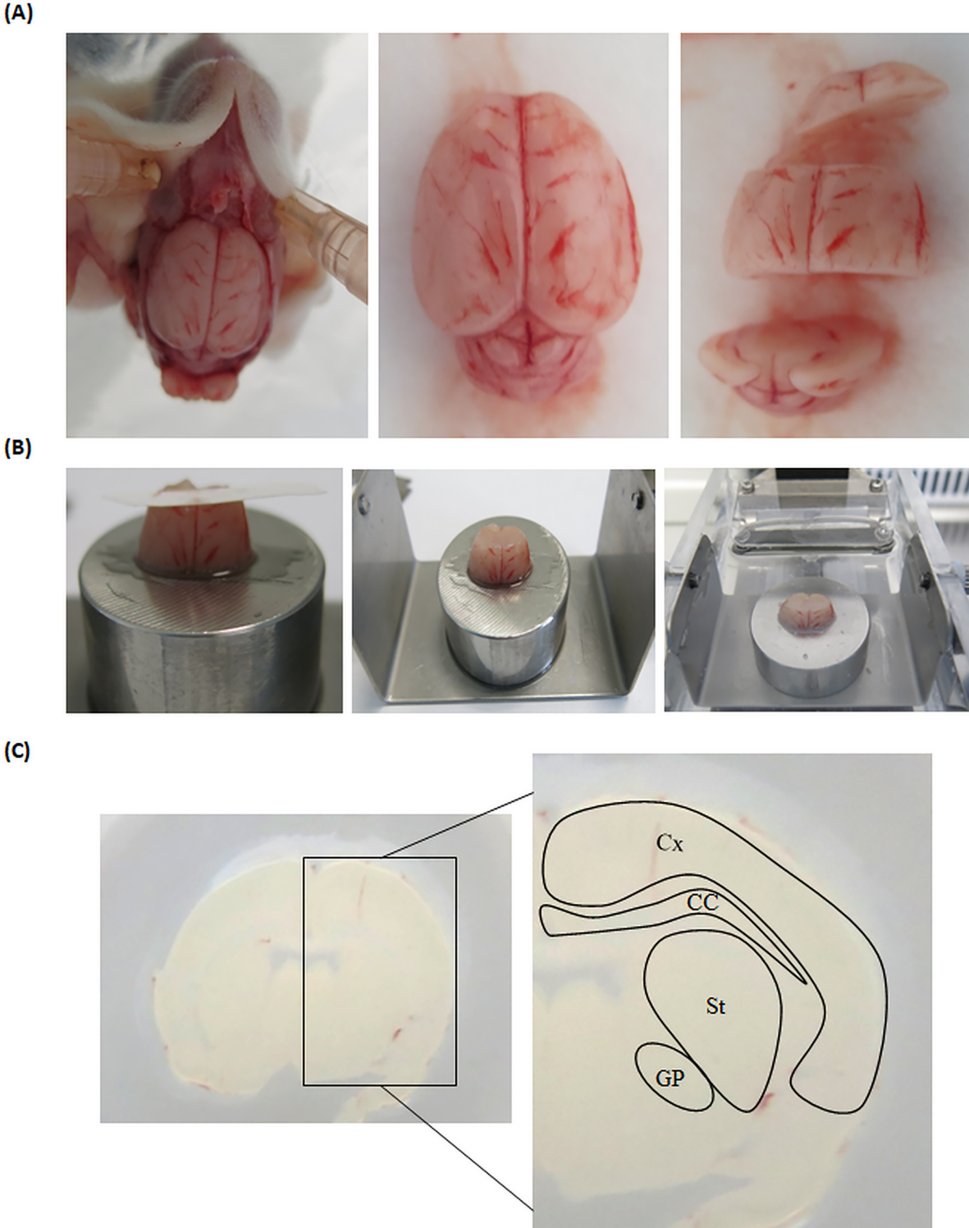
Protocol to obtain coronal HD organotypic slices. (A) The head must be dissected quickly and carefully to preserve brain structure. (B) The brain was glued onto the chuck of an aqueous solution-cooled vibratome, and 400μm thick coronal brain sections were cut and collected. (C) Brain slices were disposed on a 0.4 μm membrane insert. Coronal slices display different brain areas such as cortex (Cx), striatum (St), globus pallidus (GP) and corpus callosum (CC) associated with HD. (N = 25).

Organotypic cultures were prepared as previously described [31] with some modifications. Albinos wild-type Sprague–Dawley rats from the SCAHU (Service commun d’animalerie hospitalo-universitaire, N°49007002, Angers University, France) were used. Postnatal 5 (P5-6) and 9–11 (P9-11) days old Sprague–Dawley pups were used and their efficacy to generate the *ex vivo* model of HD was compared. Animals were rapidly sacrificed after anesthesia, by intraperitoneal injection of 80 mg/kg of ketamine (Clorketam 1000, Vetoquirol, Lure, France) and 10 mg/kg of xylazine (Rompum 2%, Bayar Health Care, Kiel, Germany). Brains were removed and rapidly dissected before being glued onto the chuck of a vibratome cooled with a bath of Gey’s balanced salt solution supplemented with 6.5 mg/L of glucose and antibiotics (Fig 1A and 1B). 400 μm thick slices were cut using a vibratome (Motorized Advance Vibroslice MA752, Campdem instruments) in different configurations to obtain a progressive degeneration of the GABAergic MSNs (Fig 1C).

Typically, about 10 slices can be obtained per brain. The first three and the last three brain slices did not contain all the main areas involved in HD and were discarded. A total of 4 different slices was obtained from each rat brain. Hemispheres were mechanically separated to culture 8 independent organotypic brain slices. Obtained slices were next transferred to 30 mm diameter semi-porous membrane inserts (Millicell-CM, Millipore, Guyancourt, France) (Fig 1C) within a 6-well plate, containing Neurobasal medium (Gibco, Life Technologies, Illkirsch, France) supplemented with 6.5 mg/L of glucose, 1mM of L-glutamine, 1x B27 supplements (Gibco, Life Technologies, Illkirsch, France) and antibiotics. Slices were incubated at 37°C and 5% CO_2._ Each slice was cultured on separated membrane to increase their survival over time. A total of about 25 rat pups and about 200 organotypic slices were necessary to perform the whole characterisation.Slice axis selection to obtain a Huntington’s disease model.

To obtain organotypic slice HD model, brains were cut with different axes to achieve an efficient sectioning of GABAergic pathway and thus the most complete dopamine- and cAMP-regulated neuronal phosphoprotein (DARPP32)- and Glutamate decarboxylase 67 (GAD67)- positive cell degeneration over time. For this analysis, three axes have been chosen: sagittal, coronal and transversal. For sagittal slices, brain hemispheres were separated and glued onto the chuck of an aqueous solution-cooled vibratome and slices were cut alongside the midline. Concerning coronal slices, cerebellum and olfactive bulbs/prefrontal cortex were cut-off, and brains were glued, on their dorsal side, onto the vibratome chuck. At last, to perform transversal slices, the underside of the brain was glued on the vibratome chuck. For each condition, 400 μm slices were performed with razor blade angle of 14°. Once the coronal slices were chosen, hemisphere were separated and cultured on different membranes to increase their survival over time.

### Histological studies

At different time points, ranging from 0 to 30 days, organotypic slices were washed with phosphate-buffered saline (PBS) (Lonza, Verviers, Belgium), fixed with 4% paraformaldehyde (PFA) (Sigma–Aldrich, St Louis, MO, USA) in PBS, pH 7.4, for 2 h and then washed three times with PBS. Finally, slices were removed from membrane inserts and stored at 4°C in PBS until use

#### Immunohistochemistry

A mouse anti-GAD67 (5 μg/mL, clone 1G10.2, Millipore SA, Guyancourt, France) and a mouse anti-DARPP32 (0.25 μg/ml, clone 15, BD Bioscience, Le Pont de Claix, France) antibodies were used to observe striatal-GP GABAergic neurons. An antibody against neuronal nuclei (NeuN) (20 μg/mL, clone A60, Merck Millipore, France) was used to observe the viability of neurons within the brain organotypic slices. Isotypic antibodies were used to control background staining.

After 2h of saturation in PBS, triton 1% (PBST), BSA 4%, and normal goat serum 10% (NGS), slices were incubated 48 h with the primary antibody diluted in 1% PBST, BSA 4% at 4°C. After washes, slices were incubated with the biotinylated anti-mouse secondary antibody (2.5 μg/mL Vector Laboratories, Burlingame, USA). Then slices were washed, and quenching of peroxidase was performed with 0.3% H_2_O_2_ (Sigma, Saint Quentin Fallavier, France) in PBS, at RT for 20 min. After PBS washes, slices were incubated with Vectastain ABC reagent (Vector Laboratories, Eurobio, Les Ulis, France) in PBS at Room temperature for 2h. Sections were then washed and revealed with 0.03% H_2_O_2_, 0.4 mg/mL diaminobenzidine (DAB, Sigma, Saint Quentin Fallavier, France) in PBS, 2.5% nickel chloride (Sigma, Saint Quentin Fallavier, France) and dehydrated before mounting.

#### Quantification of DARPP32 and GAD 67 positive fibres and cells

GAD67-positive neurons, DARPP32-positive neurons as well as NeuN-positive neurons were counted in the striatum at different time-points, from 0 until 30 days post-lesion, using Metamorph software from Molecular Devices (Roper Scientific, Evry, France). Briefly, the region of interest in the control slice (isotypic control) and the equivalent in the slice analysed were selected by taking as reference the surrounding structures (corpus callosum, hippocampus, etc…). Then, the intensity of the staining in those areas was measured. To eliminate the background the intensity measured in the control slice was subtracted to the intensity measured in the slice analysed. Intensity measured was then compared to the one measured in the intact slice (at day 0), right after brain vibrosection, which represents a 100% survival of the cells present in the organotypic brain slice. Results were presented as mean differences +/- average deviation and were calculated from 8 independent pictures taken from 6 different rats for each group.

#### Immunofluorescence

Immunofluorescence was performed using antibodies against DARPP32 (BD Transduction Laboratories, Erembodegem, Belgium). Isotypic controls were performed for each antibody. Free-floating slices were incubated in 1% PBST (Sigma–Aldrich, St. Louis, MO, USA). After pre-blocking for 4 h with 4% BSA (fraction V, PAA Laboratories, Piscataway, NJ, USA), 10% NGS (Sigma–Aldrich, St. Louis, MO, USA) in PBST, slices were incubated for 48 h at 4°C with mouse anti-DARPP32 (0.25 μg/ml) diluted in 4% BSA PBST. After three washes with PBS, the sections were incubated for 2 h with the horse affinity-purified biotinylated anti-mouse immunoglobulin G (IgG) secondary antibody (7.5 μg/ml, Vector Laboratories, Les Ulis, France) at room temperature. Then, slices were washed and incubated for 2 h with Streptavidin Fluoroprobes 488 or 547H (Interchim, Montluçon, France) diluted 1:400 or 1:1000 respectively in PBS. Finally, the sections were washed and mounted using fluorescent mounting medium (Dako, Carpinteria, CA, USA).

### Culture of neural stem cells

A lineage of human NSCs expressing green fluorescent protein (GFP), namely hNSC1 was used [32,33] and cultured as previously published [31]. Briefly, cells were cultured in a DMEM/F12 (1:1) (Glutamax, Gibco) medium supplemented with 6,5 mg/L of glucose (Sigma Aldrich), Hepes (Hepes Buffer 1M, Sigma Aldrich), 0,5% of Albumax (Gibco), 1% of N2 supplements (Gibco), 20 ng/ml of EGF and bFGF (both from R&D systems), 1% of non essentials amino acids (NEAA, Biowhitaker Lonza, Belgium) and antibiotics/antimycotics (100 U/mL penicillin, 0.1 mg/mL streptomycin, 0.25 μg/mL amphotericin B, Sigma Aldrich) on 10 μg/ml poly-d-lysine (PDL, Sigma-Aldrich, St. Louis, MO)-coated flasks.

### Injection of stem cell into organotypic slices

Two days after organotypic slice preparation, hNSCs (hNSC1) were injected into the striatum using a 22-gauge needle (Hamilton) fixed to a micromanipulator. Total injection volume consisted of 2 μl of culture media containing approximately 50.000 cells. Injections were done at 0.5 μl/minute infusion rate. The needle was left in place for 5 min to avoid the cells being expelled from the organotypic slices.

### Slice desiccation

Twenty-four hours after cell implantation, slices were dehydrated to isolate the graft by LMD. Two methods of slice water extraction were tested. Fresh organotypic slices were carefully peeled away from their culture inserts using a clean and RNaseZap (Ambion, Austin, TX, USA) treated paint-brush. The first water extraction method was based on sublimation. Slices were put on a polyethylene-naphthalate membrane-coated Petri dish (Leica Microsystems, Nanterre, France) and were then incubated at -20°C for two hours and then incubated at +30°C for one hour to induce slice water sublimation. The second method was based on alcohol dehydration. Slices were incubated for 5 min to four successive baths of ethanol (75°, 90°, 100° and 100° again). To decrease risk of RNA degradation, ethanol baths were performed at 4°C.

### Laser microdissection

Just before use, microscope and other surfaces were cleaned with RNaseZap. Immediately after slice water extraction, microdissection was carried out at room temperature with the LMD6000 microdissection system and software from Leica (Leica Microsystems, Nanterre, France) using a UV laser with a wavelength of 355 nm. Two dehydrated organotypic slices were mounted on a polyethylene-naphthalate membrane-coated Petri dish (Leica Microsystems, Wetzlar, Germany). The expression of GFP by grafted cells allowed us to identify them by fluorescence. The microdissection was achieved with settings: Power 128; Speed 1; Specimen balance 0 and 6.3x objective. Areas of approximately 13 mm^2^ of striatum were selected and collected in the cap of a 0.5 mL microfuge tube containing 70 μL of RNA Later (Life Technologies, Illkirsch, France) or lysis buffer (Macherey Nagel, Hoerdt, France). The presence of microdissected tissue samples in the cap was checked under low magnification. Striatum from two organotypic slices were pooled, centrifuged and kept à -80°C until RNA extraction. LMD was performed for no longer than 30 min per Petri dish.

### RNA integrity analysis

Identification of amplification products was performed using the Experion automated electrophoresis system (Bio-Rad Laboratories, Marnes-la-Coquette, France). All gel-based electrophoretic steps (such as sample separation, staining, de-staining, imaging, band detection and data analysis) were automatically performed to generate reproducible separation and quantitative results. For this study, DNA analysis was performed using the Experion DNA 1 K Analysis kit, which contains reagents, DNA ladder controls, microfluidic chips and other materials required to perform the DNA electrophoresis in a range varying between 25 and 1000 bp. The quantitative amount of the loaded DNA samples should range between 0.1 and 50 ng/ml. DNA fragment separation depends on the nucleotide composition in bps. Two DNA internal markers (lower (15 bp) and higher (1500 bp)) were added to allow peak alignments.

### RNA extraction and RT-qPCR

The following experimental details were performed following the guidelines of the PACEM core facility (Plate-forme d'Analyse Cellulaire et Moléculaire, Angers, France). Design of primers specific for human genes and PCR were performed as described elsewhere [34–36]. RT-qPCR was performed as previously described [34]. A housekeeping gene was tested as reference and raw data were analysed.

### Statistical analysis

Data are presented as the mean value of three independent experiments ± standard deviation (SD), unless otherwise stated. GraphPad Prism 6 was used for statistical analyses. To test normality of data, a Shapiro-Wilk test was performed. In case of a non-normal distribution, all subsequent analyses were based on log-transformed data. Then, differences between samples were determined using an analysis of variance (ANOVA) test, followed by a Tukey post-hoc test. Results were considered significant for P-values <0.05.

## Results

### Parameters to obtain optimal brain slices to study areas involved in HD

**Fig 2:**
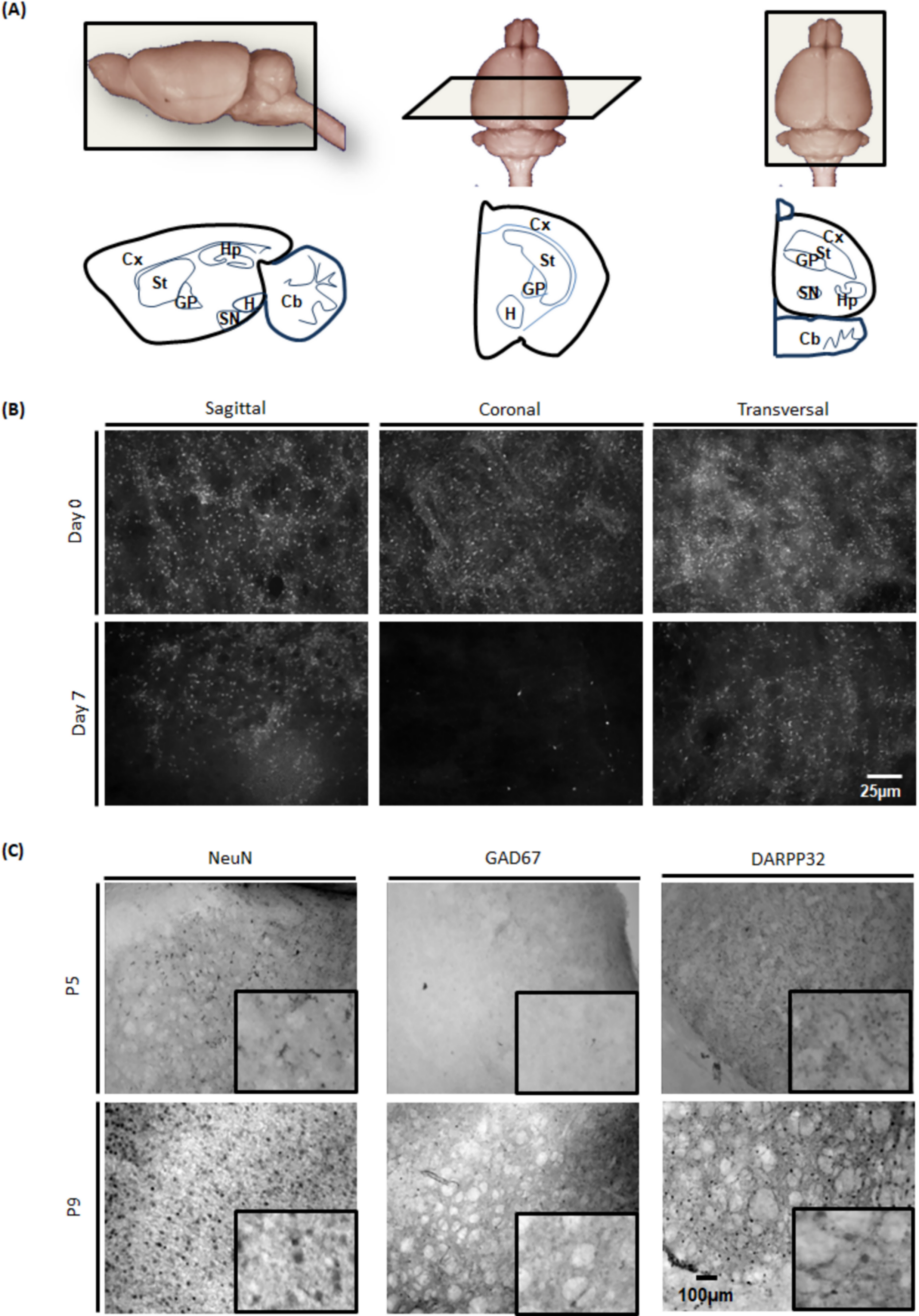
Slice plane selection to model HD and choice of pup’s age. (A) Rat's brain was cut to obtain sagittal, coronal or horizontal brain slices. In each condition, the major areas involved in HD could be observed. (B) DARPP32 was detected in brain slices by immunofluorescence at day 0 (just after sectioning) and 7 days after transversal, coronal and sagittal sections. (C) NeuN, GAD67 and DARPP32 expression was determined in coronal brain slices by immunochemistry from P5-6 and P9-11 rats immediately after sacrifice, at day 0. The maturity of brain structure in slices from P9-11 rats is closer to adult brain than slices from P5-6 rats (N = 3).

To obtain a strong mechanically induced degeneration of GABAergic MSNs, it was necessary to obtain the best slice angle to section the GABAergic neuronal pathway, particularly the striatal-GP and striatal-SNpr neuronal pathways. To this end, organotypic brain slices were prepared following three different axes. Within the three types of section obtained, the striatum, the cortex and the GP, could be easily identified (Fig 2A). The progressive degeneration of MSNs was evaluated by DARPP32 immunofluorescence. We observed that just after sectioning (at day 0), DARPP32-expressing neurons were present and observable in the whole striatum independently of the slice axis chosen (Fig 2B). In transversal and sagittal slices, DARPP32 positive cells were still highly present in the striatum after 7 days of culture. In coronal sections, a rapid reduction of DARPP32-positive cell number was observed after 4 days (data not shown), which becomes almost total after 7 days (Fig 2B). As a quick degeneration of DARPP32-positive cells was desired, coronal sections were used for the rest of this study.

To determine the appropriate age of pups to develop this novel HD model, we compared the expression of NeuN, DARPP32 and GAD67 markers in brain slices from P5 and P9 rats by immunochemistry, at day 0, right after section (Fig 2C). We observed a weaker expression of DARPP32 and GAD67 markers at P5 compared to P9, with no clear organisation of striosomes and matrix as observed in adult brains. These data confirmed that during postnatal development, the dense DARPP32 positive fibre network increases in the striatum with no major changes in the pattern of distribution. This neuronal maturation after birth is well known and detailed [37]. No significant differences were observed for NeuN marker (Fig 2C).

### Neuronal viability of organotypic brain slices

**Fig 3:**
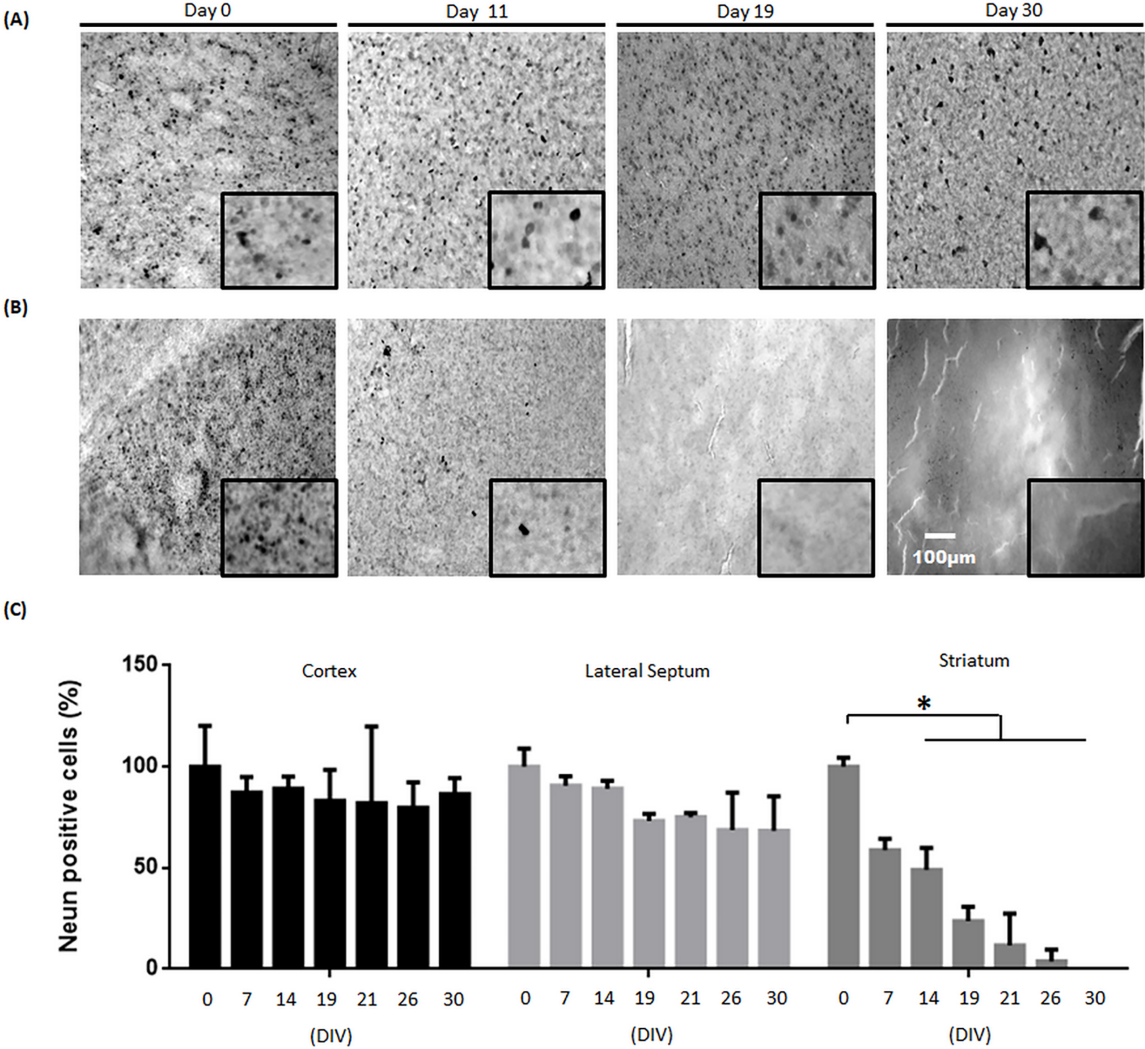
Morphology and viability of organotypic slices. Immunohistochemistry against NeuN in two brain regions: (A) cortex and (B) the striatum at day 0, 11, 19 and at day 30. (C) Quantification of NeuN-positive cells in cortex, striatum and lateral septum shows no important loss of staining in the cortex after 30 days in culture while a significant loss is observed in the striatum. (N = 4).

An immunostaining against NeuN was performed to assess neuron survival in the cortex, the lateral septum and the striatum and thus the viability of the organotypic brain slices was estimated (Fig 3). A good overall conservation of coronal organotypic slice's morphology was observed during 30 days in culture. No dramatic change of NeuN expression was observed in the cortex (Fig 3A). However, an important thinning of the striatum was observed leading to a fragility of the slice and predicting a reduction of striatal neuron viability. Indeed, within 11 days a quick depopulation of NeuN-positive cells was observed, which increased over time and became total at 30 days (Fig 3B). Visual observation was confirmed by the quantification of this marker at different time points (Fig 3C). No significant differences of NeuN-positive cell numbers were detected in the lateral septum as well as in the cortex over time. However, a significant reduction of NeuN-positive cell number was observed in the striatum. This loss reached significance at 2 weeks, after +/- 50% of cell loss, +/- 77% after 19 days and became total after 30 days, explaining the thinning of the striatum observed during the culture and confirming the neurodegeneration.

### Organotypic slice cultures display progressive degeneration of GABAergic pathway

**Fig 4:**
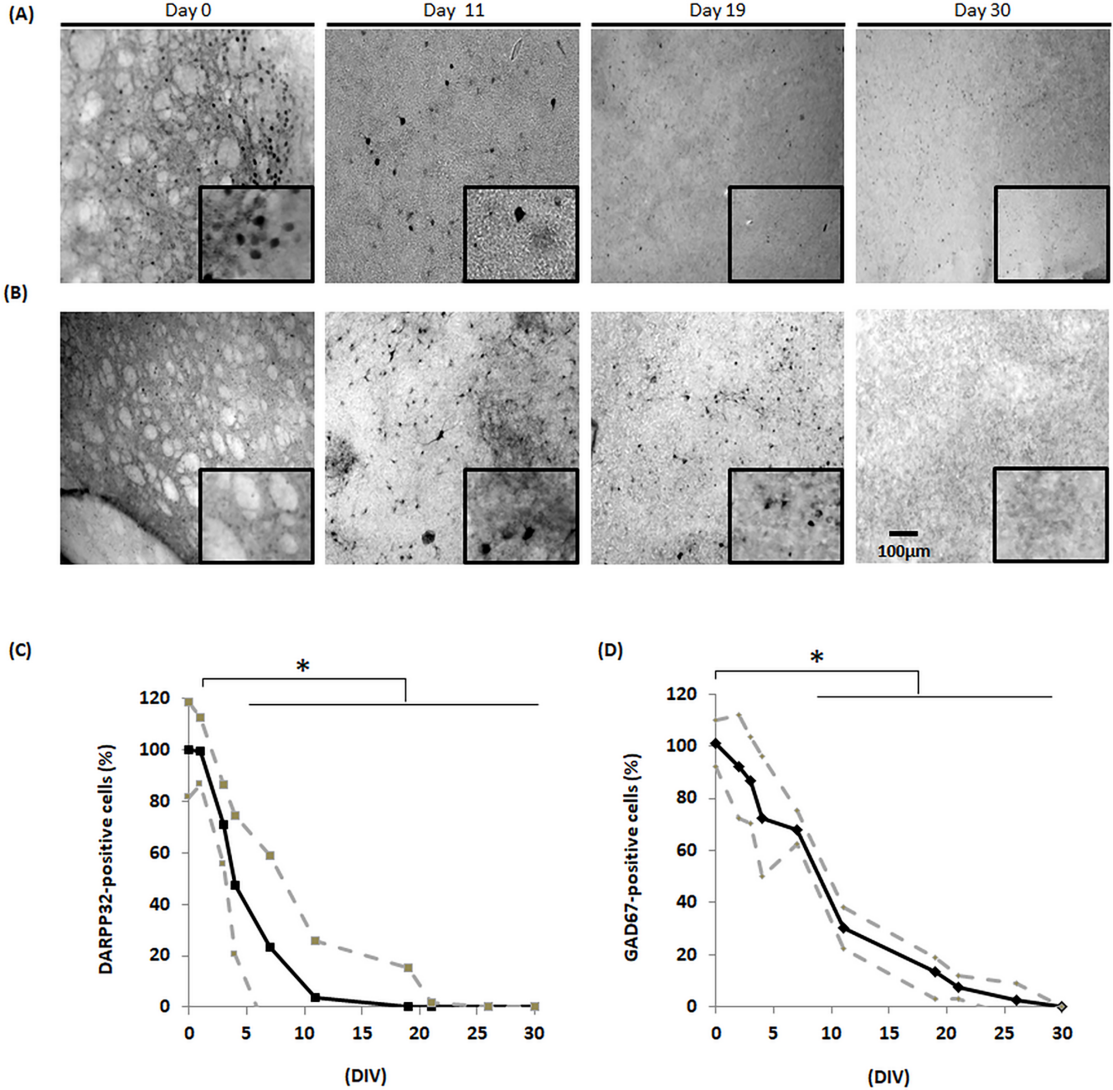
Expression of specific MSN markers. Immunohistochemistry against striatal (A) DARPP32 -positive neurons and (B) GAD67 -positive neurons at day 0, 11, 19 and 30. (C) Striatal DARPP32 -positive neuron number decreased progressively until day 19 when depopulation became total in comparison to day 0 set as 100%. (D) Striatal GAD67 -positive neuron number also decreased progressively with a 30% decrease at day 4, 45% at day 7 and 100% at day 30, compared to day 0 representing 100%. (N = 4) (SD in dotted lines).

To study the degeneration of MSNs and GABAergic neurons in the striatum, DARPP32 (Fig 4A) and GAD67 (Fig 4B) markers respectively, were analysed. The number of DARPP32-positive neurons and GAD67-positive neurons decreased over time leading to a total loss of both markers after 30 days (Fig 4A and 4B). Quantification highlighted an average DARPP32-marker loss of 25% at day 2, 50% at day 4 and 75% at day 11 and was complete by day 19, when compared to day 0 (Fig 4C). A progressive loss of GAD67 marker reached 30% at day 4, 45% at day 7 and 70% at day 11 and was almost complete by day 26 with an average of only 1% staining left compared to day 0 (Fig 4D).

### GABAergic degeneration remains similar depending on the slice section

**Fig 5:**
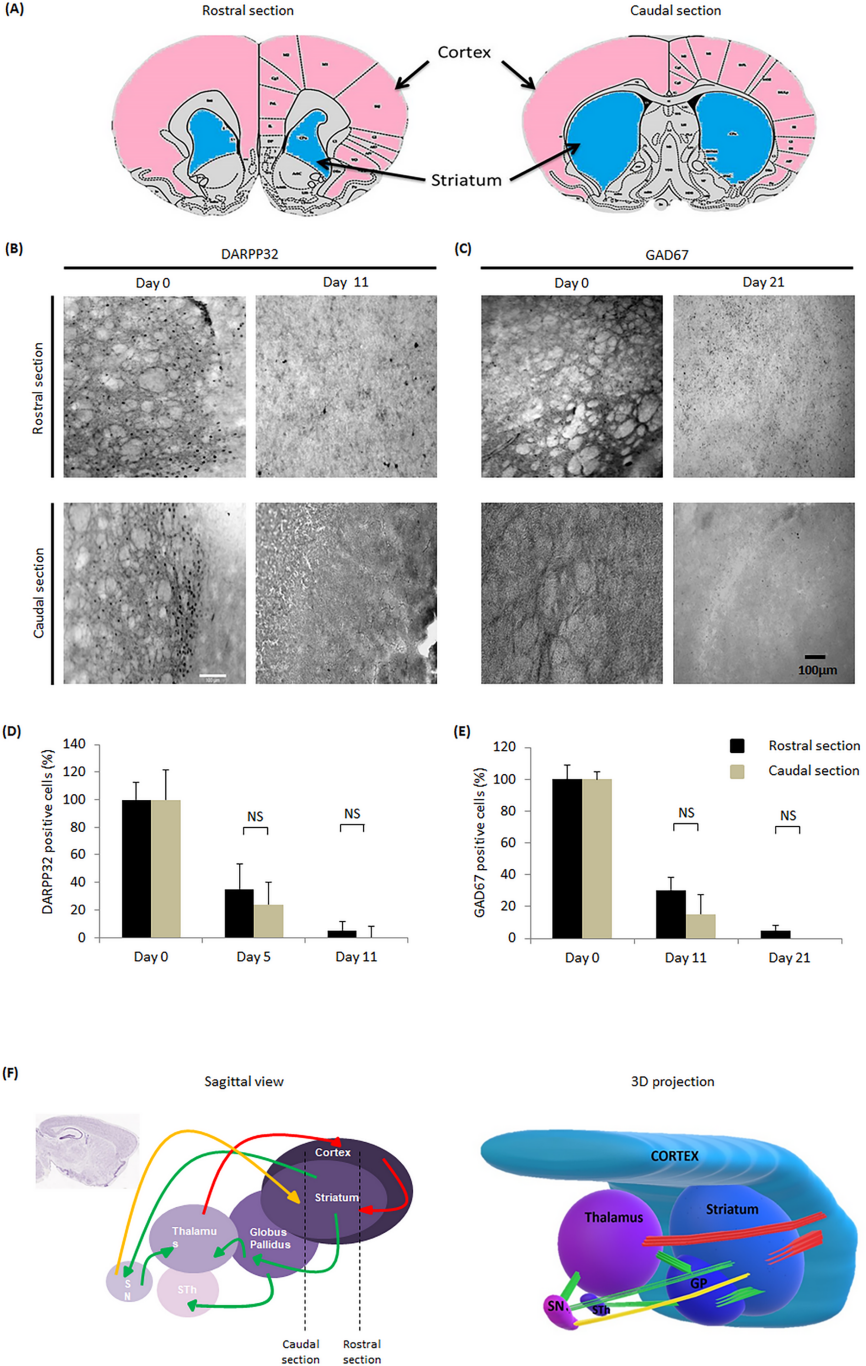
GABAergic degeneration depending on the slice section. After vibrosection, plan sections are different, and we do not consider them similarly. (A) We distinguish caudal and rostral sections of brain slices obtained after vibrosection. (B) Immunohistochemistry against striatal DARPP32 positive neurons at day 0 and day 11. (C) Immunohistochemistry against striatal GAD67 positive neurons at day 0 and day 21. The expression of those markers remained over time in rostral sections. (D) Quantifications of DARP32-positive cells over time in rostral and caudal sections. (E) Quantification of GAD67-positive cells among time in rostral and caudal sections. (F) Schematic representations of neuronal pathways observed on a sagittal view and in a 3D projection. Yellow arrows represent dopaminergic pathways. Red arrows represent Glutamatergic pathways. Green arrows represent GABAergic pathways. The top schemas represent the morphology of the brain slices obtained in this model *(taken from* *http*:*//atlas*.*brain-map*.*org*).

To further characterise this new model of HD, we compared DARPP32 and GAD67 depopulation in rostral and caudal sections (Fig 5A). DARPP32 expression is similar at Day 0 in rostral and caudal section while at Day 11, DARPP32 is still slightly visible in rostral section but not in the caudal section (Fig 5B). Similar levels of GAD67 expression were observed in both rostral and caudal sections at day 0. After 21 days, a slight staining can still be observed in the rostral section but not in the caudal section (Fig 5C). A slightly faster depopulation of DARPP32- and GAD67- positive cells was first observed. Thus, a quantification of those cells was performed at day 0, 5, 11 and 21 (Fig 5D and 5E). However, for both markers no significant differences were found between rostral sections and caudal sections over time. As observed in Fig 5F, after coronal section, for both caudal and rostral sections, the main GABAergic projections, from striatum to GP and SN, were sliced. The slight variability can be easily explained as each structure receives and sends projections through hierarchically organised, serially ordered, multi-synaptic neural pathways (Fig 5F).

### Laser microdissection on fresh organotypic slice

To evaluate the feasibility of cell transplantation and isolation by LMD. Two days after organotypic slices preparation, hNSCs (namely hNSC1) were implanted in the striatum. This cell line was chosen as hNSC1 has a strong survival yield, and expresses GFP, which allows tracking the stem cell population after implantation in the organotypic slices.

**Fig 6:**
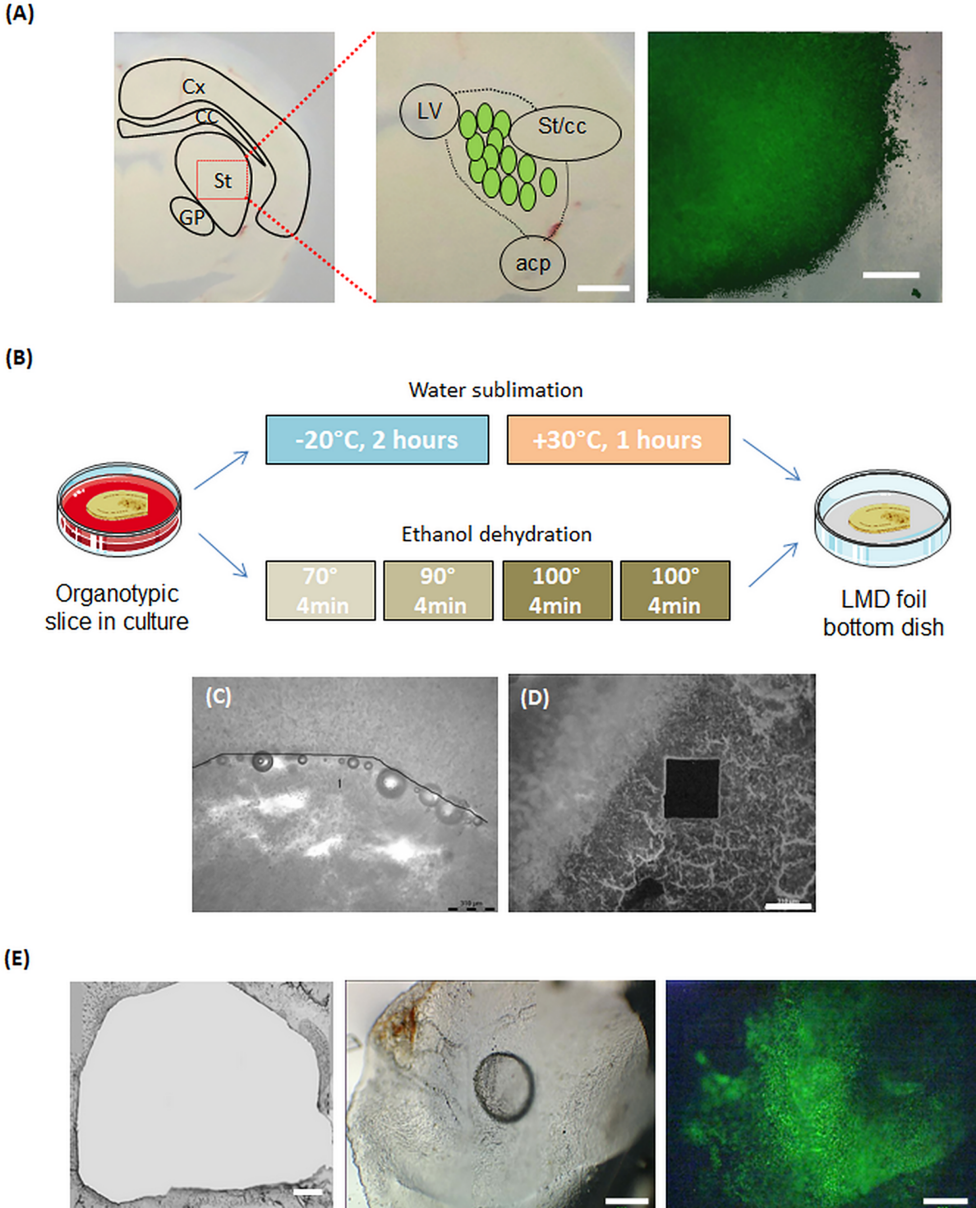
Slice dehydration protocol development to allow LMC. Entire organotypic slice with main brain areas labelled (A). Scale bar: 2mm. Striatum of organotypic slices observed by brightfield microscopy. GFP-positive hNSCs were implanted in the striatum. Striatum delimitations can be easily determined by some landmarks as the anterior commissure-posterior (ACP), the corpus callosum (CC) and the lateral ventricule (LV). Scale bar is 300μM. (B) The 1^st^ protocol was based on water sublimation after 2h incubation at -20°C followed by 1h at 30°C. The 2^nd^ protocol was based on ethanol dehydration. Four 4 min successive baths of ethanol were performed, to induce a complete desiccation of the slice. The slices were then put on a foil bottom dish to perform LMD. (C) After water sublimation, the slice was still too hydrated blocking laser dissection. (D) After ethanol dehydration, a clean laser dissection could be performed. Scale bar: 300μm (E) Organotypic slices after LMD observed by brightfield microscopy. A clear-cut of the whole striatum was performed. Collected samples observed by brightfield microscopy and by fluorescent microscope. GFP-positive NSCs can easily be observed in the collected striatum. Scale bar: 300 μm. (N = 3).

The grafted area could be easily delimited within the organotypic slices by using the lateral ventricle, the anterior commissure-posterior and the corpus callosum to identify the striatum (Fig 6A). Moreover, implanted hNSCs could be easily observed under the LMD due to their GFP expression. This allows an easy isolation of the grafted cells performed using slice morphology and GFP observation (Fig 6A).

### Slice desiccation protocol

To be able to isolate grafted cell from host tissue by LMD, slice desiccation needed to be performed first. Twenty-four hours after cells implantation, LMD was performed on organotypic slices to isolate grafted hNSCs. First, LMD was tested on fresh 400μm organotypic slices. The laser was ineffective due to the high-water content of the slice (data not shown). Water extraction from the organotypic slices was then performed to allow a clean cut of the graft. Water extraction based on water sublimation was first tested (Fig 6B). After sublimation, slices could be cut with LMD, but they got distorted, particularly at their edges. Furthermore, still too much water was retained within the slices, which made the cut difficult to perform (Fig 6C). Alcohol desiccation was then tested to remove any trace of water in the slice (Fig 6B). This method allows performing a clean microdissection of the slice (Fig 6D).

Grafted hNSCs isolation was performed following the morphological landmarks of the organotypic slices: corpus callosum, lateral ventricle and anterior commissure-posterior (Fig 6A) and using the GFP expression of implanted cells. Samples exhibited an average size of 13 mm^2^ (Fig 6E, left panel) and were collected in Eppendorf caps (Fig 6E, middle panel) in which it was still possible to observe the implanted GFP expressing cells. (Fig 6E, right panel).

### hNSCs mRNA integrity and stability after laser microdissection

Transplanted cell mRNA integrity and stability was assessed after LMD. Grafted organotypic slices were collected and treated following the two water extraction methods in RNA Later buffer or lysis buffer to determine which protocol is most efficient for hNSCs RNA extraction and to conserve hNSCs RNA integrity. Slices dehydrated by alcohol and sublimation showed a concentration of 17.9 ng/ml and 16.2 ng/ml respectively when samples were collected in RNA Later buffer. On those samples, a total quantity of 200 ng of mRNA was obtained. Concerning samples collected in lysis buffer, a concentration of mRNA close to 0.2 ng/ml was found after both dehydration protocols.

**Fig 7:**
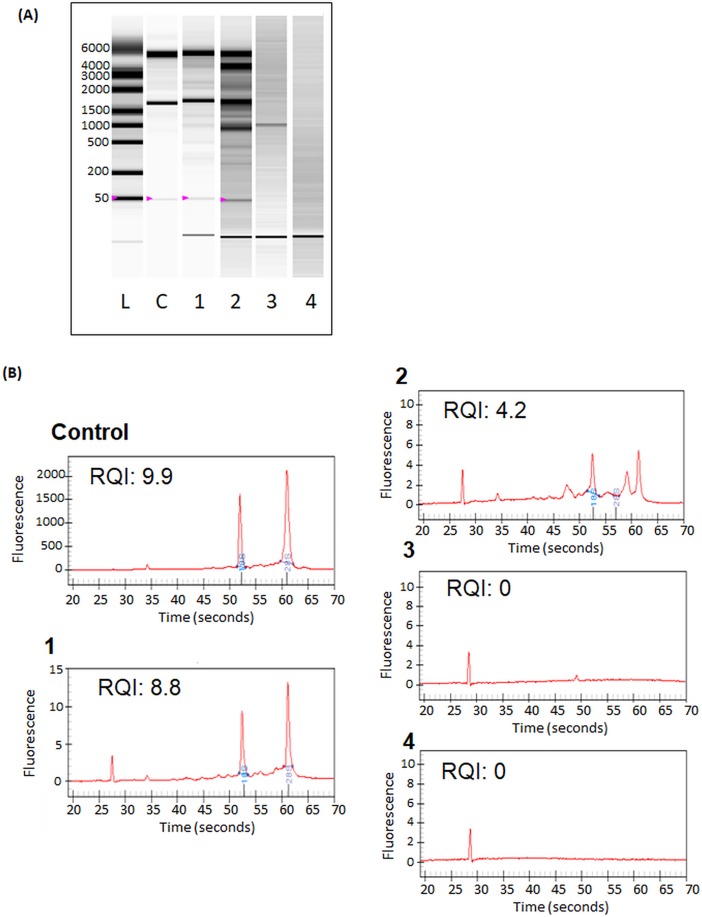
mRNA quality analysis. (A) Experion gel electrophoresis analysis. The only electrophoresis close to the control one is the alcohol treatment + RNA Later buffer conditions. (B) Electropherogram generated by Experion system of the different samples. The only high RNA quality indicator (RQI) was obtained with the alcohol treatment + RNA Later buffer condition. Conditions: L: Ladder, C: Positive control made of in vitro NSCs, 1: alcohol treatment + RNA later buffer, 2: alcohol treatment + lysis Buffer, 3: water sublimation + RNA Later buffer and 4: water sublimation + lysis Buffer. (N = 3).

Gel electrophoresis analysis demonstrated that RNA quality of alcohol-based water extraction sample (Fig 7A, column 1 and 2) was better than sample dehydrated by sublimation (Fig 7A, column 3 and 4). mRNA extracted and analysed from *in vitro* cultured hNSCs, using the same protocol used for the organotypic slice isolated tissue, served as a positive control for mRNA integrity and quality analysis. In sample collected after alcohol-based water extraction with RNA later buffer (column 1) two clear bands at 5Kb and 1.7Kb were observed corresponding to ribosomal 28S and 18S RNAs respectively. They represent +/- 80% of mammalian RNAs, highlighting the fact that the RNA collected in this condition is high quality. A very low band at 5bp (indicated by purple arrows) represents the 5s RNA. This band can only be detected in high-quality RNA samples. Plus, the band detected is very faint which highlights the fact that mRNAs are not degraded. In the alcohol-based water extraction with lysis buffer condition (column 2), a higher number of bands was observed demonstrating the lower quality of collected RNA. At last, in both samples collected after slice water sublimation (column 3 and 4), no clear bands were detected, which means that all RNAs have been degraded during sample process. This gel electrophoresis also indicated that samples collected in RNA Later buffer (Fig 7A, column 1 and 3) give better results than samples collected in lysis buffer (Fig 7A, column 2 and 4). The generated mRNA profile (Fig 7B) indicated that samples collected after alcohol-water extraction obtained a RNA quality indicator (RQI) of 8.5 +/- 0.4 in RNA Later buffer (Fig 6B–1) and a RQI of 4.2 +/- 0.13 when collected in lysis buffer (Fig 6B–2). The samples collected after slice water sublimation gave a RQI of 0 with both buffers (Fig 6B–3 and 4). In comparison positive-control mRNA gave a RQI of 9.87 +/- 0.3. For further experiment, we thus selected the protocol involving alcohol-based water extraction with sample collected in RNA Later buffer as the electrophoresis and RQI obtained are the closest to the control.

### mRNA expression analysis of host and grafted cells

The feasibility of analyzing grafted cell mRNA expression and host mRNA expression after LMD was assessed. LMD was performed on slices grafted with hNSCs as well as slices that did not receive any graft to ensure that all the primers used were human specific and that no cross-reaction could be obtained with rat tissue. Then, RT-qPCR was performed on all the collected samples (Table 1) to evaluate the *in situ* expression of a heat-shock protein (HSPb1) or of growth factors (GDNF and VEGFA).

**Table 1:** Results of a RT-qPCR performed on organotypic slice cells after LMD expressed in Ct values. In rat tissue only, rat GAPDH can be detected while all human mRNA was absent (A). In slices grafted with NSCs, human GAPDH, VEGFA and HSPB1 were detected as well as the rat GAPDH. It demonstrated that grafted human NSCs mRNA can be analysed independently from host mRNA using specific primers. A means no expression detected. (N = 3).

	Rat Tissue	Grafted NSCs
Human Glyceraldehyde-3-phosphate dehydrogenase (GAPDH)	A	21.9 ± 0.3
Human Glial cell line-derived neurotrophic factor (GDNF)	A	A
Human Vascular endothelial growth factor A (VEGFA)	A	29.4 ± 0.4
Human Heat shock protein beta-1 (HSPB1)	A	23.9 ± 0.4
Rat GAPDH	25.8 ± 4	20.9 ± 0.08

Human GAPDH (house-keeping gene), GDNF, VEGFA and HSPB1 were not detected (Absent, A in the table) in the non-grafted organotypic slices (Table 1) highlighting the fact that selected primers are human specific and do not recognise rat mRNAs. After NSCs injections in organotypic slices, human GAPDH, VEGFA and HSPB1 were detected between 21.9 ± 0.3 and 29.4 ± 0.4 cycles demonstrating that human tissue repair factors can be specifically detected and quantified in small grafts of human cells in rat tissue after LMD. However human GDNF was not detected in rat tissue or human grafted cells. In parallel, rat GAPDH mRNA was detected after 25.8 ± 4 cycles in non-grafted tissue and after 20.9 ± 0.03 cycles in NSCs grafted organotypic slices. Indeed, after LMD in grafted organotypic slices, both human and rat cells were collected (Table 1). Thus, grafted cells as well as host cells mRNA expression can be specifically analysed in this model.

## Discussion

Coronal brain organotypic slices were chosen for this study since different brain regions could clearly and easily be identified as cortex, striatum, GP, cerebellum, hippocampus and SN. Furthermore, those slices can be used to observe and study dopaminergic, GABAergic and cholinergic pathways as well as brain capillaries [34]. They can be cultured for around a month providing a powerful model to study HD. This *ex vivo* HD model could also allow studying the response from the neurogenic stem cell-niches present in the striatum and hippocampus. Furthermore, coronal section allows studying and characterizing the interactions between different neuronal populations in the GP, the striatum and the cortex, all of which are involved in HD. We here showed the feasibility of grafting stem cells in brain slices and specifically analysing gene expression of host and grafted cells. In this way, this model will be particularly interesting to perform screening of new therapeutic approaches on host GABAergic MSNs among other pathways and of grafted cells. In a similar manner it could be used to evaluate the effect of growth factors or biomaterials directly added onto slices or in the media [38,39] as previously reported by our group with organotypic model of Parkinson’s disease [31,34]. Other groups also used the advantages of organotypic slices to study HD trans-neuronal propagation [40] or new potential targets in the treatment of HD [41]. Furthermore, organotypic models have been widely used to study graft and host interactions [42] or grafted cell migration within the host tissue [43] or the effect of neurotrophic factors as BDNF and GNDF on cell survival [44] among others.

Organotypic slices obtained from P5 pups displayed a weaker expression of GABAergic MSN markers DARPP32 and GAD67, compared to slices obtained from P9 rats. We assumed that P5 pups may still have an immature striatum and we then chose to use only P9 to P11 rats for our experiments. Substantial changes to neuronal and glial organisation between organotypic slices from young and adolescent rodent have already been reported within the neocortex [45]. It is established that tissue from embryonic or post-natal donors are more plastic, they conserve a better morphology as well as a better survival rate [46]. Consequently, only P9-11 rats will be used to obtain a more stable and reproducible model. However, a young rat brain doesn’t represent the same level of maturity than an adult human brain. Nevertheless, in contrast to sporadic Parkinson’s disease, HD is not an age-related disease. In the majority of cases, symptoms appear after 40–50 years, but it has been reported that 2% of children in HD families had onsets of symptoms before the age of 10 [47]. In that case, the *ex vivo* HD model could be very useful.

To preserve brain slice viability, a serum containing media was used only during the first 3 days to conserve a strict minimum media and avoid any variability in slice survival related to serum. Indeed, it has been reported that serum containing media may induce a strong degeneration of neurons in slice cultures [48,31] while in studies in which organotypic slices were cultured in a serum-free media with B27 supplements similar to our culture conditions, a long-term viability of neurons was observed [49]. With our protocol, *ex vivo* brain slices viability was maintained up to 4 weeks. It has previously been reported that an addition of brain-derived neurotrophic factor (BDNF) in organoid culture media results in an increase of slice survival [50]. However, we decided not to use BDNF in our culture media as it was also reported that it induces an increase of neuronal survival and facilitates neurotransmitter release of dopaminergic and GABAergic neurons [51]. Plus, our goal was to conserve a strict minimum media.

This novel HD model involves GABAergic MSN degeneration due to the mechanical transection of neuronal pathways between the striatum, the SN and the GP. Indeed, the rodent striatum is made of +/- 10% of interneurons and of +/- 90% of projection neurons. The latter, all GABAergic, send connections to the SN as well as the internal and external GP [52]. The interneurons can be divided into 2 categories: large spiny neurons that use acetylcholine as a neurotransmitter and the small- to medium-sized GABAergic interneurons. We assume that when rodent brains were sliced in the coronal axis, GABAergic projection neurons were cut within the striatum, which leads to retrograde and anterograde neuronal degeneration. Indeed, major neuronal pathway projects from the striatum, which is in the rostral region of the brain to areas localized in the caudal region of the brain, including GP and SN. Thus, when coronal slices are performed on the rodent brain, both striatal-GP and striatal-SN neuronal pathways are severely damaged. While, in the case of a sagittal orientation, the slice is performed along the neuronal pathway inducing a less severe injury. A sagittal sectioning induced a maximal loss of +/- 50% of DARPP32-positive cells when this loss reached +/- 100% after a coronal sectioning. During the protocol development, the main aim was to cut the striatal-GP and striatal-SN neuronal pathways, which would lead to a GABAergic MSN degeneration, which, in our opinion, is the key to develop a simple *ex vivo* model of HD.

We followed MSNs population during a 4-week period after coronal sectioning and noted a loss of 50% of this population after 3 days which became total after 19 days compared to an intact slice. In parallel, a reduction of GABAergic markers GAD67 was measured for 4 weeks. The decrease of GAD67-positive cells reached 46,7% at day 5 and became total after 4 weeks. This difference of degeneration dynamics can be explained in part by the fact that the striatal interneurons may not be affected by this degeneration. Moreover, GAD67 may be a stronger marker than DARPP32 as GAD67 is present in both terminals and the cell body. Furthermore, GAD67 is required for normal cellular functioning, unrelated to neurotransmission [53]. In this model, 10% of GAD67-expressing cells were still detected at day 19, while no DARPP32-positive cells were present in the striatum. At the same time point we observed 23% of NeuN-positive cells which may be the sum of those GAD67-positive cells plus the large aspiny neurons present in the striatum.

As stated before, HD is characterised by a selective vulnerability with dysfunction followed by death of the MSNs. First HD organotypic models were prepared from wild-type rodents and GABAergic neuron loss was then obtained by injection of neurotoxins such as kainic acid, quinolic-acid or 3-nitropropionic acid, that can be all used at different concentrations and different exposure times. All those parameters must be taken into consideration and can lead to heterogeneity in the results [54]. Moreover, it has been previously shown that the use of neurotoxins can interfere with other drugs used during surgery such as the anaesthetic ketamine [55] [56]. In our model, GABAergic MSN degeneration is due to mechanical cutting of neuronal pathways between the striatum and SN and the GP. Thus, no neurotoxins are necessary leading to less variability between protocols. Moreover, our *ex vivo* model induces a significant and selective MSN depopulation in 4 days, which allows the development of an early model of the disease. Indeed, Vonsattel *et al* reported a loss of 50% of caudate neurons in neuropathology grade I and 95% in grade IV human HD [57]. In our model, these grades are reached after +/- 5 days and +/- 11 days respectively. Thus, it offers a window of 25 days after a grade I, or 19 days after grade IV disease, to study new therapeutic approaches.

This simple and rapid model can offer a real advantage compared to *in vivo* animals in which at least 40 days are necessary to obtain an HD-like phenotype [58]. In general, to obtain organotypic models of HD made directly from transgenic mice such as R6/2, it is necessary to wait for 15 to 21 weeks of age before clear HD symptoms, and pathological patterns occur [15,16]. At this age, it is quite tricky to obtain viable organotypic cultures. However, our model of HD is only based on the depopulation of GABAergic MSNs, MSNs, through probably a different mechanism than in HD patients in the discussion. Thus only the cellular aspect of HD can be studied, in detriment of the genetic component of the pathology [59]. In contrast, the R6/2 transgenic mice organotypic slices show some genetic background as well as HTT inclusions but less neurodegeneration. Thus, genetic models may be more interesting to study HD phenotype as HTT inclusion while our model may be more interesting to study neurodegeneration and neuroprotection/repair approaches. More recently, an organotypic HD model was developed form normal slices transfected with HD-polyQ plasmids or with DNA constructs derived from the human pathological *HTT* gene [60–62]. However, slice transfection involves high technology equipment and a skilled operator as organotypic brain slices are delicate and frequently become damaged during the preparative stages [13]. The model described in this article is low-cost, simple, rapid to perform, study and analyse, making it interesting for new therapeutic approach screening.

To study gene expression and assess normal or pathologically altered cells and tissue a method involving LMD with RT-qPCR was here developed. Optimal tissue section thickness for LMD is 4–15 μm [63], but some experiments were already made with slices up to 200μm with UV Laser Cutting [64]. In this report, 400 μm organotypic slices were successfully cut, to further isolate grafted cells from surrounding host tissue and analyse them by RT-qPCR. This LMD technique was performed not only on coronal but also on sagittal slices (not shown). The main advantage of the mRNA isolation protocol is its simplicity. Indeed, it was previously reported that RNA quality decreases with increased complexity of the tissue preparation protocol [65]. Also, in our protocol organotypic slices were incubated in ethanol and it has been reported that ethanol conserves tissue morphology and RNA integrity [66]. Other protocols of LMD on organotypic slices imply performing a supplemental cut of the organotypic slices with a cryostat to obtain 10–20μm thick slices [67,68], while in this study mRNA expression was analysed directly after the LMD of 300–400μm thick tissue. Thus, this protocol allows having easily reproducible results. Still, it is important to note that the thickness of an organotypic slice requires that we use the laser set at a high power associated to a low magnification, which reduces the precision of the dissection. Therefore, this LMD protocol is more adapted to zones of minimum 5 mm^2^ for microdissection.

In this study, one sample is composed of around 50.000 human grafted cells in the rat tissue, which represent a batch of cells in an important enough number to study the mRNA by RT-qPCR or DNA chip and DNA-microarray. Indeed, it has been reported that with an optimized protocol gene analysis by RT-qPCR can be performed on a very small cell population, from 1000 to 10 cells [69] or even on single cell [70]. However, our aim is to study an entire cohort of grafted cell, so single cell analysis is not applicable here. It has previously been reported that this line of hNSCs have a good survival and integration after implantation in brain organotypic slices [34] as well as in vivo [71]. We here demonstrated that mRNAs extracted from tissue dissected with LMD were very high quality and in a high enough concentration to study multiple gene expression. Moreover, grafted human cell samples can be analysed independently from rat cells, using human specific primers that do not cross-react with rat. Thus, this technique allows analysing the expression of different genes associated with growth factors or proteins involved in grafted cells behavior (proliferation, differentiation etc…). Furthermore, rat tissue mRNA can be analysed separately with rat-specific primers, in samples with or without cell grafts to determine the reaction of the host tissue to the cell implantation (neuroprotection/repair).

In summary, organotypic slices have been widely used to model neurological pathologies including Parkinson's disease [31,34], brain stroke and cerebral ischemia among others [13]. This novel model represents a promising tool to quickly and efficiently test innovating treatments for HD using stem cells and biomaterials [72].

## Conclusion

In this study, we developed and characterised a relevant *ex vivo* model of HD reproducing the GABAergic degeneration that can be used to study the early stage of the pathology and the relevance of new and innovative treatments. Moreover, we developed a new protocol allowing LMD to be performed on 400μm thick organotypic slices with the extraction of good quality mRNA from a small region in these slices. This model and LMD protocol can be easily used to study and obtain a comprehensive knowledge of the grafted cell behavior, host tissue responses and host cells/implanted cells interactions.

## Abbreviations

BSA: Bovine Serum Albumin
DARPP32: Dopamine- and cAMP-Regulated neuronal PhosphoProtein of 32kDa
GABA: gamma-Aminobutyric acid
GAD67: Glutamate decarboxylase 67
GFP: green fluorescent protein
GP: Globus Pallidus
GBSS: Gey’s Balanced Salt Solution
HD: Huntington's disease
*HTT*: Huntingtin gene
HTT: Huntingtin protein
LMD: Laser Microdissection
MEM: Minimum Essential Medium Eagle
mRNA: messenger ribonucleic acid
MSN: Medium Spiny Neuron
NeuN: Neuronal Nuclei
NGS: Normal Goat Serum
NSCs: Neural Stem Cells
NPs: Neural Progenitors
PBS: Phosphate Buffered Saline
PFA: Paraformaldehyde
PolyQ: Polyglutamine
RT-qPCR: real-time quantitative PCR
*SNpr*: substantia nigra *pars reticulata*
